# Cholesterol-Dependent Energy Transfer between Fluorescent Proteins—Insights into Protein Proximity of APP and BACE1 in Different Membranes in Niemann-Pick Type C Disease Cells

**DOI:** 10.3390/ijms131215801

**Published:** 2012-11-26

**Authors:** Bjoern von Einem, Petra Weber, Michael Wagner, Martina Malnar, Marko Kosicek, Silva Hecimovic, Christine A. F. von Arnim, Herbert Schneckenburger

**Affiliations:** 1 Department of Experimental Neurology, Ulm University, Helmholtz Str. 8/1, 89081 Ulm, Germany; E-Mails: bjoern.von-einem@uni-ulm.de (B.E.); christine.arnim@uni-ulm.de (C.A.F.A.); 2 Institut für Angewandte Forschung, Hochschule Aalen, Anton-Huber Str. 21, 73430 Aalen, Germany; E-Mails: petra.weber@htw-aalen.de (P.W.); michael.wagner@htw-aalen.de (M.W.); 3 Division of Molecular Medicine, Rudjer Boskovic Institute, Bijenicka 54, 10000 Zagreb, Croatia; E-Mails: martina.malnar@irb.hr (M.M.); marko.kosicek@irb.hr (M.K.); silva.hecimovic@irb.hr (S.H.)

**Keywords:** FRET, cholesterol, APP, BACE1, NPC, TIRFM, neurodegeneration, laurdan, generalized polarization

## Abstract

Förster resonance energy transfer (FRET) -based techniques have recently been applied to study the interactions between β-site APP-cleaving enzyme-GFP (BACE1-GFP) and amyloid precursor protein-mRFP (APP-mRFP) in U373 glioblastoma cells. In this context, the role of APP-BACE1 proximity in Alzheimer’s disease (AD) pathogenesis has been discussed. FRET was found to depend on intracellular cholesterol levels and associated alterations in membrane stiffness. Here, NPC1 null cells (CHO-*NPC1*^−/−^), exhibiting increased cholesterol levels and disturbed cholesterol transport similar to that observed in Niemann-Pick type C disease (NPC), were used to analyze the influence of altered cholesterol levels on APP-BACE1 proximity. Fluorescence lifetime measurements of whole CHO-wild type (WT) and CHO-*NPC1*^−/−^ cells (EPI-illumination microscopy), as well as their plasma membranes (total internal reflection fluorescence microscopy, TIRFM), were performed. Additionally, generalized polarization (GP) measurements of CHO-WT and CHO-*NPC1*^−/−^ cells incubated with the fluorescence marker laurdan were performed to determine membrane stiffness of plasma- and intracellular-membranes. CHO-*NPC1*^−/−^ cells showed higher membrane stiffness at intracellular- but not plasma-membranes, equivalent to cholesterol accumulation in late endosomes/lysosomes. Along with higher membrane stiffness, the FRET efficiency between BACE1-GFP and APP-mRFP was reduced at intracellular membranes, but not within the plasma membrane of CHO-*NPC1*^−/−^. Our data show that FRET combined with TIRF is a powerful technique to determine protein proximity and membrane fluidity in cellular models of neurodegenerative diseases.

## 1. Introduction

Brain cholesterol is the main component of cellular membranes in the central nervous system (CNS) and plays an essential role in regulation of membrane fluidity, and thereby, structural integrity and functional specificity at various cellular locations is important for neuronal development and survival [1–4], synapse maturation and optimal synaptic activity [5,6]. As a consequence, brain cholesterol homeostasis is essential for the physiological function of the CNS, and imbalanced brain cholesterol levels are linked to neurodegenerative diseases including Huntington’s disease (HD) [7,8], Parkinson’s disease (PD) [9–11], Alzheimer’s disease (AD) [12–15] and Niemann–Pick type C disease (NPC) [16–18].

The amyloid hypothesis of AD pathogenesis favors amyloid-β peptide (Aβ), a product of the sequential proteolytic cleavage of the transmembrane amyloid protein precursor (APP) by β-site APP-cleaving enzyme (β-secretase or BACE1) and γ-secretase, as a trigger for AD [19–22]. The proteolytic processing of APP by β- and γ-secretase occurs predominantly in cholesterol-rich microdomains, so called lipid rafts, at intracellular membranes [23–27]. Several risk factors for AD are associated with cholesterol metabolism, including dyslipidaemia, coronary artery and cerebrovascular disease [28]. Apart from age, the ε4 allele of Apolipoprotein E (ApoE), a cholesterol carrier, is the major risk factor for sporadic AD [29–32]. Several independent studies have shown that changes in cholesterol level lead to altered APP processing by BACE1 and, subsequently, altered levels of APP fragments (Aβ and secreted APPβ (sAPPβ)) [27,33–35]. Niemann–Pick type C disease (NPC) is a lysosomal storage disorder caused by mutations in the genes *NPC1* or *NPC2*. The resulting dysfunction in proteins involved in intracellular cholesterol transport and homeostasis leads to intracellular accumulation of cholesterol and sphingolipids in late endosomes and lysosomes [18,36,37]. NPC symptoms include progressive ataxia, dystonia and dementia. An increasing number of studies in cell culture, as well as in animal models, revealed parallels in the molecular mechanisms of NPC and AD pathology [38–41]. Like AD, NPC goes along with impaired cholesterol homeostasis, altered APP metabolism and abnormal tau phosphorylation [42].

In previous experiments, we and others demonstrated that APP processing by BACE1, and subsequently sAPPβ secretion, is increased with rising intracellular cholesterol levels, whereas sAPPα secretion is reduced [33]. Cholesterol depletion by MβCD leads to the opposite effect [33–35,43,44]. Using EPI-illumination microscopy (for detection of whole cells) combined with FRET measurements, we have recently shown [33] that APP-mRFP and BACE1-GFP proximity was decreased at intracellular compartments associated with increased cholesterol level and membrane stiffness, whereas no changes could be observed at the plasma membrane, as measured by FRET experiments under TIRFM conditions (further abbreviated as TIRET). Increased APP cleavage by BACE1 and sAPPβ secretion was likewise found in CHO-*NPC1*^−^*^/^*^−^ cells [35]. As these cells are also enriched in intracellular cholesterol, we used them to extend our previous study regarding membrane stiffness and altered proximity of APP and BACE1 upon increased cholesterol levels.

## 2. Results and Discussion

### 2.1. CHO-*NPC1*^−/−^ Cells are Enriched in Total, as well as, Vesicular Cholesterol

**Figure 1:**
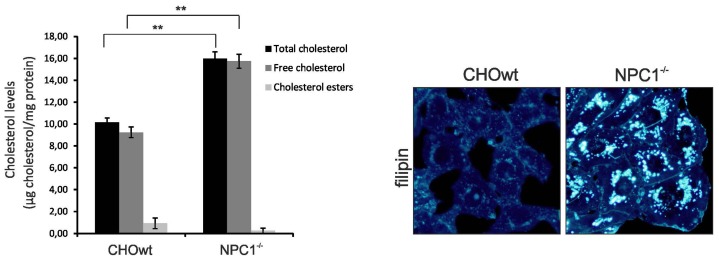
CHO-*NPC1*^−^*^/^*^−^ cells show increased levels of free and total cholesterol and vesicular accumulation of free cholesterol. Cholesterol levels (total cholesterol, free cholesterol and cholesterol esters) and free cholesterol accumulation were analyzed in CHO-*NPC1*^−^*^/^*^−^*vs.* CHO-WT cells. Cholesterol levels were determined in cell lysates by AmplexRed Cholesterol Assay (Invitrogen, Darmstadt, Germany) (**left panel**). Note that total cholesterol in CHO-*NPC1*^−^*^/^*^−^ cells is mainly comprised of free cholesterol, with only negligible amounts of esterified cholesterol. Shown are the mean and SEM of three independent experiments. Statistical analysis was performed using Student’s *t*-test: **p < 0.01. Filipin staining shows punctuate accumulation of free cholesterol in CHO-*NPC1*^−^*^/^*^−^ cells compared to CHO-WT cells (**right panel**).

As we wanted to compare the measurements with our recently published data of cholesterol loaded and depleted glioblastoma astrocytoma U373 cells [33], we first performed cholesterol level measurements of CHO-WT and CHO-*NPC1*^−^*^/^*^−^ cells (Figure 1). As expected, CHO-*NPC1*^−^*^/^*^−^ cells had a significantly higher free and total cholesterol levels compared to CHO-WT. To further visualize the distribution of free cholesterol within the cells, we performed filipin staining (Sigma-Aldrich). As described for NPC, we found highly increased vesicular distribution of free cholesterol in CHO-*NPC1*^−^*^/^*^−^ cells compared to CHO-WT (Figure 1).

Interestingly, we found that CHO-*NPC1*^−^*^/^*^−^ cells had a cholesterol level comparable with cholesterol depleted U373 glioblastoma cells (Supplementary Table S1). This can be explained by the high cholesterol levels in astrocytes. Astrocytes supply neurons with cholesterol, as neurons do not produce enough cholesterol on their own.

### 2.2. *NPC1*^−/−^ Cells Display Higher Membrane Stiffness at Intracellular Membranes

To further analyze differences in membrane stiffness associated with altered cholesterol levels, we used the previously described method of wide-field fluorescence microscopy for measuring membrane dynamics of living cells based on 6-dodecanoyl-2-dimethylamino naphthalene (laurdan) staining and generalized polarization (GP) measurements [45]. As reported previously [45,46], GP represents a normalized value for the difference of fluorescence intensity in two spectral bands, with the first one being predominant in a rather stiff-, and the second one in a more fluid environment. Using TIRF and EPI-illumination, we were able to distinguish between alterations of membrane stiffness at the plasma membrane and intracellular membranes.

**Figure 2:**
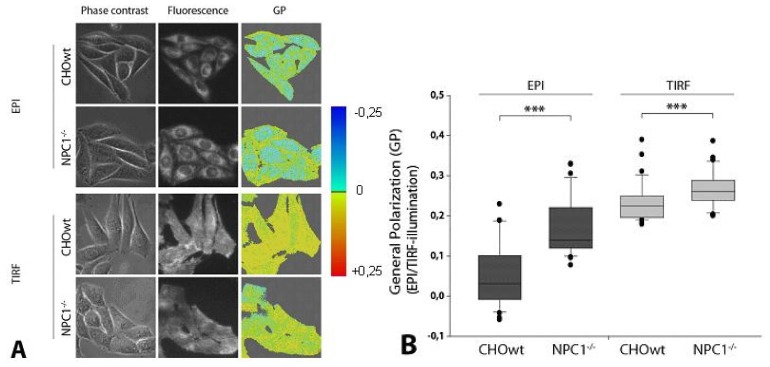
CHO-*NPC1*^−^*^/^*^−^ cells show significantly increased membrane stiffness. (**A**) from right to left: generalized polarization (GP) values (with a color code from blue to red corresponding to fluid-stiff), corresponding fluorescence intensities (recorded at λ ≥ 420 nm) and phase contrast images of CHO-WT and CHO-*NPC1*^−^*^/^*^−^ cells, incubated with laurdan as a function of intracellular cholesterol amount for whole cells (EPI-illumination) and plasma membranes (TIRF illumination) at T = 24 °C; (**B**) GP at the plasma membranes of CHO-*NPC1*^−^*^/^*^−^ cells (TIRF) is only slightly increased compared to CHO-WT cells (*n* = 32; ***p < 0.001). In contrast, GP is strongly increased at intracellular compartments of CHO-*NPC1*^−^*^/^*^−^ cells compared to CHO-WT (EPI) (*n* = 32; ***p < 0.001).

As observed before in cholesterol loaded U373 cells, we found only slightly increased stiffness of the plasma membrane as measured by TIRFM. However, using EPI-illumination of whole cells, we found highly increased membrane stiffness at intracellular membranes in CHO-*NPC1*^−^*^/^*^−^ cells compared to CHO-WT (Figure 2). This is most likely due to the massive cholesterol load of intracellular compartments in *NPC1*^−^*^/^*^−^ cells (Figure 1). These results are in line with our earlier measurements of U373 cells, where cholesterol loading increased membrane stiffness and also occurred mainly at intracellular compartments, but not at the plasma membrane [33].

### 2.3. APP and BACE1 Proximity is Decreased in *NPC1*^−/−^ Cells, but Not at the Cell Surface

**Figure 3:**
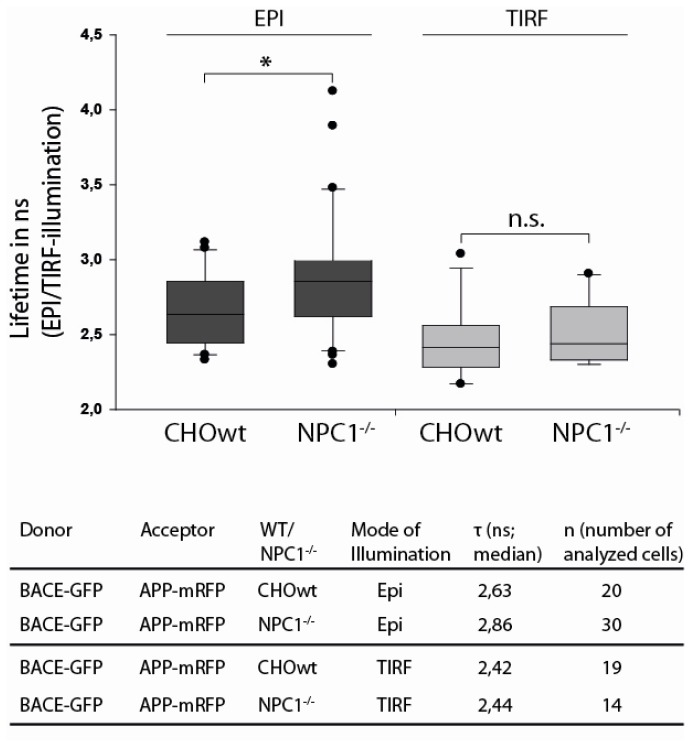
Fluorescence lifetime of GFP for probing GFP-RFP proximity in BACE1-GFP and APP-mRFP double stably transfected CHO cells. Lifetimes at plasma membranes, as shown by TIRF, are not significantly altered. Under EPI-illumination, BACE1-GFP lifetimes of CHO-*NPC1*^−^*^/^*^−^ cells are significantly higher compared to CHO-WT cells (Mann-Whitney rank sum test: * *p* = 0.039).

To address the question whether APP and BACE1 proximity is altered at the cell surface or at intracellular membranes due to an increased cholesterol level in CHO-*NPC1*^−^*^/^*^−^ cells, we combined EPI or TIRF microscopy with fluorescence lifetime measurements, as described earlier [33]. Following excitation by picosecond laser pulses, fluorescence decrease of the donor BACE1-GFP was mono-exponential with the lifetimes depicted in Figure 3.

Non-radiative energy transfer from a donor to an acceptor molecule, according to the Förster mechanism [47], is often determined from fluorescence lifetime experiments, since the lifetime τ of an excited molecular state corresponds to the reciprocal of the sum of its deactivation rates, including fluorescence (*k*_F_), internal conversion (*k*_IC_), intersystem crossing (*k*_ISC_) and intermolecular energy transfer (*k*_ET_). If τ is the lifetime of a donor molecule in presence of, and τ_0_ in absence of an acceptor molecule, the difference 1/τ −1/τ_0_ reflects the energy transfer rate *k*_ET_ from the donor to the acceptor. For dipole-dipole interaction, this rate is proportional to R^−6^, with R representing the intermolecular distance, which generally is smaller than about 10 nm. An increase of the fluorescence lifetime τ of the donor can be related to a decrease of *k*_ET_, and therefore, to a decrease of donor-acceptor (in this case BACE1-GFP and APP-mRFP) proximity.

In agreement with previous results [33] (and in contrast to an APP-BACE positive control [33]), we found no significant changes in donor lifetime and, therefore, in APP-BACE1 proximity at the cell surface as measured by TIRFM. However, under EPI illumination, we observed a significant increase of donor lifetime in CHO-*NPC1*^−^*^/^*^−^ cells compared to CHO-WT (Figure 3). This indicates that APP and BACE1 come less often into close proximity in CHO-*NPC1*^−^*^/^*^−^ cells compared to CHO-WT and that that enhanced membrane stiffness due to cholesterol enrichment is associated with decreased proximity of APP and BACE1 in CHO-*NPC1*^−^*^/^*^−^ cells.

Data on how cholesterol influences APP processing are quite inconsistent. Age of the cells, as well as cell lines used for the experiments, seem to influence the results. However, consistent with our previous findings, most data from *in vitro* and *in vivo* experiments show increased APP β-cleavage upon cholesterol increase, and reduced β-cleavage and increased α-cleavage upon cholesterol depletion [27,34,44,48–51]. To understand how BACE1-cleavage of APP is increased, while at the same time APP-BACE1 proximity is reduced, seems to be quite challenging. But one has to keep in mind that, besides its influence on cellular membranes, altered cholesterol levels induce complex alterations in cell metabolism and might influence different membranes to different extent. It is possible that, in some compartments, APP-BACE1 proximity is decreased, whereas in others, APP processing by BACE1 is increased. Although in CHO-*NPC1*^−^*^/^*^−^ cells APP and BACE1 were shown to accumulate in the same compartments (transferrin receptor-positive vesicles) [52], their routing to and out of these compartments could be quite different, which in turn could result in their overall decreased proximity within the cell. Indeed, we showed recently a strong colocalization of APP, and not BACE1, with early endosomal marker EEA1, and a marked accumulation of APP *C*-terminal fragments (APP-CTFs), and not BACE1, within late endosomes in CHO-*NPC1*^−^*^/^*^−^ cells [52]. Regarding transport and endocytosis rates and mechanisms, there is little data about alterations upon cholesterol enrichment, but it might lead to accelerated turnover and endocytosis of APP and BACE1 in lipid rafts [53], thereby promoting APP processing by BACE1. We have previously reported that APP localization is shifted to lipid rafts in CHO-*NPC1*^−^*^/^*^−^ cells [54]. Another explanation might be the direct impact of cholesterol environment on BACE1 activity. In living cells, BACE1 seems to require intact rafts for activity [23], and BACE1 outside rafts seems to be inactive. Cholesterol enrichment, as found in CHO-*NPC1*^−^*^/^*^−^, might change raft composition and size, thereby altering BACE1 activity. Additionally, FRET efficiency is strongly dependent on the spatial orientation of donor and acceptor molecules and, in particular, orientation of donor and acceptor dipole moments. Besides cholesterol, lipid rafts are also enriched in large molecules, like sphingolipids, and proteins that project beyond the phospholipid bilayer and might influence the spatial orientation of the donor and acceptor molecules. Therefore, we cannot completely rule out that increased membrane stiffness and altered raft composition due to cholesterol enrichment might promote APP-BACE1 interaction, but at the same time, influence GFP-RFP FRET efficiency due to sterical hindrance.

## 3. Experimental Section

### 3.1. Expression Constructs

We utilized previously described APP695-rfp and BACE1-gfp expression constructs [55–57].

### 3.2. Cell Culture and Stable Transfection

Chinese hamster ovary wild type (CHO-WT) cells and CHO-*NPC1*^−^*^/^*^−^ cells (kindly provided by Dr. Daniel Ory) were maintained as described previously [52]. Cell lines stably expressing APP-mRFP and BACE1-GFP were established by co-transfection using Lipofectamine LTX (Invitrogen, Darmstadt, Germany) and selection in the media supplemented with an appropriate selective antibiotic.

Both cell lines were seeded on microscope slides and maintained in DMEM (Gibco) supplemented with 10% fetal bovine serum (FBS) and 1x penicillin/streptomycin (P/S). Cells were incubated in 5% CO_2_ and 37 °C for 24 h to 36 h before analysis.

### 3.3. Cholesterol Quantification

CHO-WT and CHO-*NPC1*^−^*^/^*^−^ cells were plated in 6-well plates and grown for 48 h in 10% FBS DMEM/F12 medium until confluent. Cells from each well were collected and lysed in CoIP lysis buffer (50 mM Tris, pH 7.6, 150 mM NaCl, 2 mM EDTA, 1% NP40) containing protease inhibitor cocktail (Roche Applied Science). Protein concentration in cell lysates (mg protein/mL) was determined with the DC Protein Assay (BioRad). The protein concentration in cell lysate is proportional to the number of cells collected for lysis. Concentration of free cholesterol, cholesterol esters and total cholesterol in cell lysates (μg cholesterol/mL) was measured using Amplex Red Cholesterol Assay Kit (Molecular Probes), following the manufacturer’s instructions. Cholesterol concentration was normalized according to protein concentration of the matching sample. Thus, cholesterol levels were expressed in μg cholesterol/mg protein.

### 3.4. Filipin Staining

Filipin (Sigma Aldrich) staining was done as previously described [58]. The cells were mounted and were viewed by fluorescence microscope (Olympus BX51).

### 3.5. Laurdan Staining and GP Measurements

The hydrophobic membrane marker 6-dodecanoyl-2-dimethylamino naphthalene (laurdan) was obtained from Molecular Probes (Göttingen, Germany) and prepared as a 2 mM stock solution in ethanol. For GP measurement, cells were grown on microscope object slides for 48 h prior to rinsing with Earl’s balanced salt solution (EBSS) and incubation with laurdan (8 μM in culture medium) for 60 min at 37 °C. GP measurement and calculation was performed as described previously [45] using the following equation: GP = (I_440_ − I_490_)/(I_440_ + I_490_), with I_440_ and I_490_ representing the fluorescence intensities measured at 440 nm and 490 nm, respectively.

### 3.6. Microscopic Imaging and FRET Analysis

Microscopic experiments were performed with a picosecond laser diode emitting light at 470 nm (LDH 470 with driver PDL 800 B, Picoquant, Berlin, Germany; average power 1 mW, pulse duration 70 ps, repetition rate 40 MHz) as the excitation source of GFP. This laser diode was adapted to a fluorescence microscope (Axioplan 1, Carl Zeiss Jena, Germany) with either a multimode fiber and a dichroic mirror (reflecting light at wavelengths λ ≤ 510 nm) for EPI-illumination, or with a single mode fiber (Point Source, kineFlex, Southampton, UK) for illumination by an evanescent electromagnetic field (penetration depth around 130 nm) with a specific device for total internal reflection (TIR) fluorescence microscopy [59]. In the first case, whole cells were illuminated, whereas in the second case, selective illumination of the plasma membranes and adjacent cellular sites occurred. Using a purpose-made optical setup [33,45,60], one could easily switch between the two excitation modes. For measuring fluorescence decay kinetics of single cells, a long pass filter transmitting light above 515 nm and a time-gated image-intensifying CCD camera system (Picostar HR 12; LaVision, Göttingen, Germany) with a time resolution of 200 ps were used, as described in detail elsewhere [61]. Fluorescence decay kinetics of individual cells were recorded by shifting the time gate of 200 ps over an axis of 20 ns. Since the fluorescence intensity of mRFP was very low, all fluorescence was related to GFP.

For GP evaluation, cells incubated with laurdan (see above) were used and exposed to a laser diode emitting light at 391 nm (LDH 400 with driver PDL 800 B; Picoquant, Berlin) upon EPI-illumination and a laser diode at 375 nm (LDH 375; Picoquant, Berlin) upon TIR illumination. Simultaneous use of both laser diodes facilitated parallel measurements under maintenance of similar extinction coefficients [46]. Fluorescence spectra of single cells were recorded at λ ≥ 420 nm with a polychromator and an image intensifying detection unit (IMD 4562, Hamamatsu Photonics, Ichino-Cho, Japan) [62] fixed on top of the microscope. In addition, fluorescence images were recorded at 450 ± 20 nm and 490 ± 20 nm using appropriate interference filters and an electron multiplying (EM-)CCD camera (DV887DC, ANDOR Technology, Belfast, U.K.) with Peltier cooling and a sensitivity below 10^−16^ W/pixel.

## 4. Conclusion

Combination of FRET and TIRF (TIRET) (in comparison with FRET under EPI illumination) is an appropriate and very selective technique to examine molecular interactions in the plasma membrane of living cells, since illumination by an evanescent electromagnetic field arising at a cell-substrate surface is limited to less than 100 nm. By combining FRET with EPI illumination, we were able to confirm previously observed cholesterol-associated alterations in proximity of AD relevant proteins APP and BACE1 at intracellular compartments, in contrast to the plasma membranes. In the future, alterations in protein proximity may be further localized within the cells by fluorescence lifetime imaging microscopy (FLIM), where the same experimental setup can be used. This may permit more detailed information about processes involved in the formation of neurodegenerative disorders and other diseases.

## Supplementary Information
