# Supplementary material for: Cholesterol-Dependent Energy Transfer between Fluorescent Proteins—Insights into Protein Proximity of APP and BACE1 in Different Membranes in Niemann-Pick Type C Disease Cells
Source: Int J Mol Sci. 2012 Nov 26;13(12):15801–12. doi: 10.3390/ijms131215801 (PMC3546662; doi:10.3390/ijms131215801)
Supplement: Supplementary file 1 [file ijms-13-15801-s001.pdf]

# Supplementary Information

**Table S1.** Total cholesterol levels: analyzed in CHO-*NPC1*$^{-/-}$, CHO-WT, untreated U373, cholesterol loaded U373 and cholesterol depleted U373 cells. Total cholesterol levels were determined in cell lysates by AmplexRed Cholesterol Assay (Invitrogen, Darmstadt, Germany).

| Cells | Total cholesterol levels (µg cholesterol/mg protein) |
|---|---|
| CHO-WT | 10.0 |
| **CHO-*NPC1*$^{-/-}$** | **16.0** |
| U373 untreated | 31.9 |
| U373 cholesterol loaded (100µM) | 60.8 |
| **U373 cholesterol depleted (4mM MβCD)** | **16.9** |

© 2012 by the authors; licensee MDPI, Basel, Switzerland. This article is an open access article distributed under the terms and conditions of the Creative Commons Attribution license (http://creativecommons.org/licenses/by/3.0/).
